# Optimization of neuron-specific interfering peptides targeting GABA_B_ receptor downregulation for proteolytic stability for conferring neuroprotection in a mouse model of cerebral ischemia

**DOI:** 10.3389/fphar.2025.1576884

**Published:** 2025-04-28

**Authors:** Mohammad Hleihil, Musadiq A. Bhat, Thomas Grampp, Dietmar Benke

**Affiliations:** ^1^ Institute of Pharmacology and Toxicology, University of Zurich, Zurich, Switzerland; ^2^ Neuroscience Center Zurich, University and ETH Zurich, Zurich, Switzerland

**Keywords:** GABAB receptor, interfering peptide, cerebral ischemia, neuron-specific targeting, RVG blood brain barrier shuttles (BBBpS), middle cerebral artery occlusion (MCAO), excitotoxicity, neuroprotection

## Abstract

**Background:**

Cerebral ischemia triggers a cascade of detrimental events, leading to brain damage mainly due to the over-excitation of neurons. Currently, clinically applicable neuroprotective treatments to stop progressive neuronal death remain elusive. The GABA_B_ receptor, crucial for neuronal inhibition, is a promising target for neuroprotection because it inhibits neuronal over-excitation which otherwise leads to excitotoxic death. However, ischemic conditions impair GABA_B_ receptor function by downregulating the receptors via pathologically altered trafficking events. Previously, we developed interfering peptides to inhibit the interaction of GABA_B_ receptors with key interacting proteins, leading to the pathological downregulation of the receptors. These interfering peptides restored GABA_B_ receptor expression and function, resulting in reduced excitability and death of neurons in *in-vitro* and *ex-vivo* models of cerebral ischemia. However, the interfering peptides were not effective *in-vivo* because of their limited proteolytic stability after systemic application.

**Methods/results:**

Here, we aimed to render three interfering peptides resistant to proteolytic degradation by replacing natural L-amino acids by D-amino acids. Additionally, we optimized a blood brain barrier shuttle (BBBpS) sequence derived from the Rabies virus glycoprotein (RVG) that mediates neuron-specific uptake and blood-brain barrier crossing of these interfering peptides. By optimizing the peptides, we developed stable, neuron-specific interfering peptides that successfully restored GABA_B_ receptors expression and prevented neuronal death following excitotoxic stress in cultured neurons. *In vivo* testing in the middle cerebral artery occlusion (MCAO) mouse model of cerebral ischemia demonstrated the neuroprotective activity of the optimized peptides by a significantly reduced infarct size.

**Conclusion:**

These findings confirm the potential of these peptides as neuroprotective agents and emphasize the importance of proteolytic stability of peptide drugs for their successful *in-vivo* application.

## 1 Introduction

Stroke remains one of the leading causes of death and long-term disability worldwide, primarily resulting from the sudden disruption of cerebral blood flow (Feigin et al., 2017; Katan and Luft, 2018; Collaborators, 2021). Cerebral ischemia accounts for most cases of stroke and triggers a complex cascade of pathological events, including oxidative stress, excitotoxicity, neuroinflammation, and angiopathy, ultimately leading to progressive cerebral damage and neuronal death (Qin et al., 2022; Shehjar et al., 2023). Despite significant advances in acute stroke management to restore reperfusion, effective neuroprotective strategies to mitigate progressive loss of neurons remain elusive for clinical application (Grupke et al., 2014; Amantea and Bagetta, 2017). This unmet need underscores the urgency of developing novel neuroprotective approaches to limit progressive neuronal death after stroke.

One promising target for neuroprotection in cerebral ischemia is the GABA_B_ receptor, a heterodimeric metabotropic G-protein-coupled receptor composed of the subunits GABA_B1_ and GABA_B2_, that plays a crucial role in mediating prolonged neuronal inhibition in the central nervous system (Bettler et al., 2004; Bassetti, 2022; Benke et al., 2024). GABA_B_ receptors have been implicated in neuroprotection by attenuating excitotoxic damage via reducing neuronal excitability (Lal et al., 1995; Jackson-Friedman et al., 1997; Babcock et al., 2002; Liu et al., 2015; Hleihil et al., 2021; Bhat et al., 2023; Benke et al., 2024). However, ischemic conditions disrupt GABA_B_ receptor function by promoting their degradation, resulting in reduced neuronal inhibition and heightened neuronal vulnerability (Guetg et al., 2010; Maier et al., 2010; Terunuma et al., 2010; Hleihil et al., 2021). This downregulation of GABA_B_ receptors is mediated by specific protein-protein interactions resulting in dysregulated trafficking of the receptors that exacerbate neuronal damage and thus provides potential targets for the development of novel neuroprotective interventions. Pathological over-excitation of neurons is associated with ER stress, which induces the expression of the pro-apoptotic transcription factor CHOP. Upregulated CHOP binds to the GABA_B2_ subunit in the ER to prevent their heterodimerization with the GABA_B1_ subunit and thereby the exit of the functional heterodimeric receptor complex from the ER (Maier et al., 2014). This mechanism inhibits the supply of new receptors and eventually downregulates GABA_B_ receptor expression because constitutively degraded receptors are not replaced. In a second pathway, constitutively internalized GABA_B_ receptors are dephosphorylated at GABA_B2_(S783) by the protein phosphatase 2A (PP2A). This inhibits fast recycling of the receptors (Terunuma et al., 2010; Hleihil et al., 2022). Subsequent phosphorylation of GABA_B1_ by calcium calmodulin-dependent kinase II β (CaMKIIβ) triggers the sorting of the receptors to the lysosomal degradation pathway (Guetg et al., 2010; Zemoura et al., 2019). So far, we have developed interfering peptides that inhibit the interaction of GABA_B_ receptors with the key regulatory proteins causing the receptor downregulation: CaMKII (Balakrishnan et al., 2023), PP2A (Hleihil et al., 2022), C/EBP homologous protein (CHOP (Bhat et al., 2022)) and the transmembrane E3 ubiquitin ligase MARCH1 (Bhat et al., 2025). These interfering peptides restored GABA_B_ receptor expression, normalized neuronal excitability and inhibited progressive neuronal death after an ischemic insult *in-vitro* and *ex-vivo*.

A significant limitation of peptide-based drugs is their potential for rapid degradation by proteolytic enzymes after systemic application, which can prevent or significantly reduce their efficacy (Tugyi et al., 2005; Di, 2015). In this study, we aimed at optimizing three of our interfering peptides, which are proteolytically degraded in blood serum within a view minutes, for proteolytic stability by exchanging natural L-amino acids by unnatural D-amino acids. This approach is an easy way to render peptides proteolytic stable but at the risk of reducing or loosing their activity (Tugyi et al., 2005; Di, 2015).

There are numerous cell-penetrating peptide and blood brain barrier peptide shuttle sequences available, but the vast majority lack cell-type specificity. As we aim to specifically target neurons in the brain, we used a peptide sequence derived from the Rabis virus glycoprotein (RVG peptide), which is selectively taken up by neurons (Kumar et al., 2007; Balakrishnan et al., 2023; Hleihil and Benke, 2024) and traverse the blood-brain barrier to deliver cargo into the brain (Kumar et al., 2007). However, a main limitation of this peptide sequence is its lengths of 41 amino acids, which leads to interfering peptides that are overly long, often costly and challenging to synthesize. Therefore, we also aimed to optimize the sequence of the BBBpS with respect to length and proteolytic stability.

Optimizing the RVG peptide and our specific interfering peptides resulted in proteolytic stable peptides. The interfering peptides retained their neuron-specificity and their activity to restore GABA_B_ receptor expression as well as their neuroprotective activity in cultured neurons after excitotoxic stress. Finally, all three interfering peptides considerably reduced the infarct size in the middle cerebral artery occlusion (MCAO) mouse model of cerebral ischemia. These findings confirm our previous *in-vitro* and *ex-vivo* observations and demonstrate the efficacy of the peptides *in-vivo*.

## 2 Materials and methods

### 2.1 Antibodies

The following Antibodies were used for this study: rabbit GABA_B2_ directed against the N-terminus of GABA_B2_ (affinity-purified, used for cell surface staining, 1:250 for immunofluorescence (IF); custom made by GenScript (Benke et al., 2002)), rabbit GABA_B2_ (1:500 for IF; Abcam #ab75838), rabbit NeuN (1:400 for IF, Millipore #ABN78), mouse NeuN (1:400 for IF, Millipore #MAB377), rabbit GFAP (1:1000 for IF, Abcam #ab7260). For immunofluorescence staining, secondary antibodies used were labeled with Alexa Fluor Plus 488, 555, and 647 (1:2000, ThermoFisher). Detection of the biotinylated peptides was performed with Alexa Fluor 488-streptavidin (1:2000 for IF, Jackson ImmunoResearch # 016-540-084) and Alexa Fluor 750-streptavidin (1:5000 for Western blotting, ThermoFisher #S21384).

### 2.2 Peptides

All peptides used in this study were custom synthesized by Pepmic Co., Ltd., Suzhou, China and were tagged with biotin for detection. The peptides were provided as acetate salt with a purity >95%. The peptides are given below with the respective experiments where they were used. Characters in black indicate natural L-amino acids and those in red unnatural D-amino acids.

**Table:** 

Peptide name	Sequence	Used in:
1.	YTIWMPENPRPGTPCDIFTNSRGKRASNGGGGRRRRRRRRR	Figures 1A,B
2.	YTIWMPENPRPGTPCDIFTNSRGKRASNGGGG	Figure 1A
3.	YTIWMPENPRPGTPCDIFTNSRGKRASNG G G G	Figure 1A
4.	Y T I WMPENPRPGTPCDIFTNSRGKRASNGGGG	Figure 1A
5.	YTIWMPENPRPGTPCDIFTNSRGKRASNGGGG R R R R R R R R R	Figure 1A
6.	Y T I WMPENPRPGTPCDIFTNSRGKRASNGGGGRRRRRRRRR	Figure 1A
7.	YTIWMPENPRPGTPCDIFTNSRGKRASN G G GGRRRRRRRRR	Figure 1A
8.	YTIWM P E NPRPGTPCDIFTNSRGKRASNGGGGRRRRRRRRR	Figure 1A
9.	YTIWMPENPRP G T PCDIFTNSRGKRASNGGGGRRRRRRRRR	Figure 1A
10.	YTIWMPENPRPGTPCDIFTNSRG K R ASNGGGGRRRRRRRRR	Figure 1A
11.	YTIWMPENPRPGTPCDI F T NSRGKRASNGGGGRRRRRRRRR	Figure 1A
12.	YTIWMPENPRPGTPCDIFTNSRGKRASNGGGGRRRRRRRRR	Figures 1A,B
RVG-M1-Pep L-amino acids	YTIWMPENPRPGTPCDIFTNSRGKRASNGGGGRRRRRRRRRD KDLEEVTMQLQDTPEKTTY	Figure 1C
RVG-M1-Pep D-amino acids	YTIWMPENPRPGTPCDIFTNSRGKRASNGGGGRRRRRRRRRD KDLEEVTMQLQDTPEKTTY	Figures 1C,E
RVG-M1-Pep scrambled L-amino acids	YTIWMPENPRPGTPCDIFTNSRGKRASNGGGGRRRRRRRRRQTD TYKMLDQELTTDPEEVK	Figure 1D
RVG-M1-Pep scrambled D-amino acids	YTIWMPENPRPGTPCDIFTNSRGKRASNGGGGRRRRRRRRRQTD TYKMLDQELTTDPEEVK	Figure 1D
1.	YTIWMPENPRPGTPCDIFTNSRGKRASNGGGGRRRRRRRRR	Figures 2A,B
2.	YTIWMPENRRRRRRRRR	Figures 2A,B
3.	ENPRPGTRRRRRRRRR	Figure 2A
4.	GTPCDIFTRRRRRRRRR	Figures 2A–C
5.	FTNSRGKRRRRRRRRRR	Figures 2A,B
6.	KRASNGGGGRRRRRRRRR	Figure 2A
CHOP-Pep	GTPCDIFTRRRRRRRRRLQDTPEKTTYIK	Figures 4A,D, 5
CaMKII-Pep	GTPCDIFTRRRRRRRRRSETQDTMKTGSSTNNNEEEKSR	Figures 4B,E, 5
PP2A-Pep	GTPCDIFTRRRRRRRRRFQFTQNQKKEDSKTSTSV	Figures 4C,F, 5
Ctrl-Pep	GTPCDIFTRRRRRRRRRQKFSVNTFQEKDTKSQTS	Figure 5

The interfering peptides CHOP-Pep, CaMKII-Pep and PP2A-Pep were previously identified by screening a library of synthetic peptides (15–25 amino acids long) comprising all intracellularly located amino acid sequences of GABA_B1_ and GABA_B2_ for their ability to prevent the ischemia-induced downregulation of GABA_B_ receptors by inhibiting the interaction of GABA_B_ receptor with CHOP (Bhat et al., 2022), CaMKII (Balakrishnan et al., 2023) and PP2A (Hleihil et al., 2022).

The control peptide (Ctrl-Pep) is a scrambled version of CaMKII-Pep and was used to verify the specificity of the interfering peptides in the *in-vivo* experiments. The sequence of CaMKII-Pep was selected to design the control peptide because it was the longest sequence among the three interfering peptides used in this study.

Unless otherwise stated, all peptides were used at a concentration of 10 μg/mL (corresponding to 2–2.5 μM in case of the optimized interfering peptides) for the *in-vitro* experiments and 200 μg/mouse for the *in-vivo* experiments.

### 2.3 Animals

Middle cerebral artery occlusion (MCAO) experiments were conducted using adult male and female C57BL/6J mice aged 9–12 weeks. The mice were housed in groups of up to five per cage under a standard 12-h light/dark cycle, with unrestricted access to food and water. All procedures were performed in compliance the national guidelines of the Swiss Federal act on animal protection as well as with the ARRIVE guidelines and were approved by the Zurich Cantonal Veterinary Office, Zurich, Switzerland (License ZH004/2023).

The procedure of preparation of neuron/glia co-cultures from 18 days old embryos of pregnant Wistar rats (ENVIGO, Netherlands) was approved by the Cantonal Veterinary Office Zurich (license ZH087/2022).

### 2.4 Primary neuron/glia co-cultures

All cell culture media used were from Gibco Life Technologies. The pregnant rat was deeply anesthetized with isoflurane and euthanized by decapitation. After removal of the 18 days old embryos, they were killed by decapitation and the cerebral cortex was dissected. After washing with 5 mL sterile-filtered PBGA buffer (PBS containing 10 mM glucose, 1 mg/mL bovine serum albumin and antibiotic-antimycotic 1:100 (10,000 units/ml penicillin; 10,000 μg/mL streptomycin; 25 μg/mL amphotericin B) the cortices were cut into small tissue pieces with a sterile scalpel and digested in 5 mL sterile filtered papain solution for 15 min at 37°C. Then, the tissue was washed twice with complete DMEM/FBS medium (Dulbecco’s Modified Eagle’s Medium containing 10% Fetal Bovine Serum and penicillin/streptomycin, 1:100). After adding 3–4 mL of fresh DMEM/FBS, the tissue was carefully and gently triturated. The triturated cell suspension was filtered through a 40 μm cell-strainer and finally plated at a concentration of 90,000 cells per well onto poly D-lysine coated coverslips in a 12-well culture dish. After 3–4 h of incubation at 37°C and 5% CO_2_, the DMEM medium was exchanged with freshly prepared NU-medium (Minimum Essential Medium (MEM) with 15% NU serum, 2% B27 supplement, 15 mM HEPES, 0.45% glucose, 1 mM sodium pyruvate, 2 mM GlutaMAX). The cultures were kept for 12–16 days at 37°C and 5% CO_2_.

### 2.5 Determination of peptide uptake using the ODYSSEY CLx scanner

**FIGURE 1:**
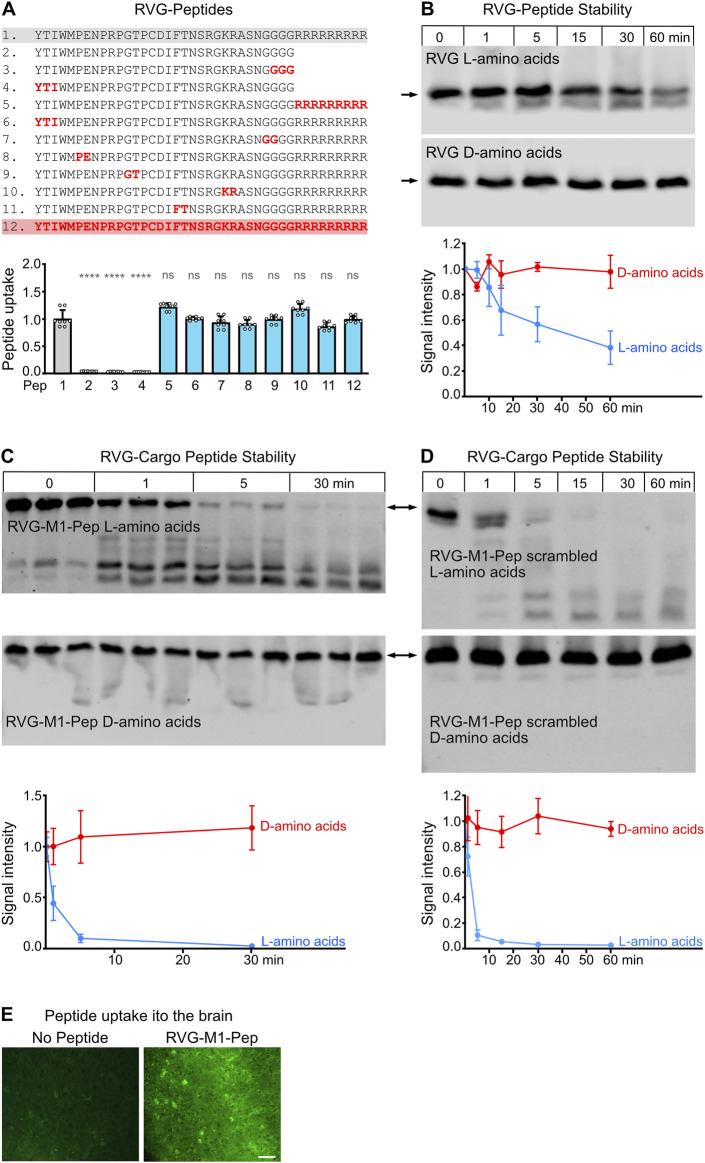
Optimization of the neuron-specific RVG peptide for proteolytic stability. **(A)** Exchange of natural L-amino acids for unnatural D-amino acids did not affect its uptake into neurons. Neuron/glia cultures were treated for 1 h with the indicated biotinylated peptides. Peptides were stained with Alexa Fluor 750 conjugated streptavidin and detected by the Odyssey CLx scanner. Top: RVG sequences tested. Red characters depict D-amino acids, black characters L-amino acids. Bottom: Quantification of peptide uptake into cultured neurons. The number on the x-axes refers to the number of the RVG peptide sequence indicated in the top panel. The unchanged original RVG sequence (1) served as control. The data represent the mean ± SD of the analysis of 8 individual wells (cultures) per sequence derived from three independent experiments. Brown-Forsythe and Welch’s one-way ANOVA followed by Games-Howell’s multiple comparisons test (ns, p > 0.05; ****p < 0.0001). **(B)** Proteolytic stability of RVG peptides composed of L-amino acids (blue) or D-amino acids (red). Peptides were added to blood serum for the indicate times and analyzed thereafter by Western blotting. Top: representative Western blots (arrows indicate the position of the undegraded peptides). Bottom: Quantification of signal intensities. Time points 0 served as control. The data represent the mean ± SD of four independent experiments. **(C,D)** Proteolytic stability of the RVG peptide with the attached cargo peptides M1-Pep **(C)** or M1-Pep with scrambled sequence **(D)**. Peptides were either composed of L-amino acids (blue) or D-amino acids (red). Peptides were added to blood serum for the indicate times and analyzed thereafter by Western blotting. Top: representative Western blots (arrows indicate the position of the undegraded peptides). Bottom: Quantification of signal intensities. Time points 0 served as control. The data represent the mean ± SD of three independent experiments and one technical replicate. **(E)** Uptake of RVG-M1-Pep into the brain. Mice were injected into the tail vein or not with RVG-M1-Pep (200 μg/mouse) and tested for the presence of the peptide in brain tissue by immunohistochemistry. The images shown were taken from the cerebral cortex and are representative for 6 mice tested (scale bar: 40 μm).

For quantifying the uptake of RVG peptides into cultured neuron/glia cells shown in Figure 1A, the cultures were grown in poly D-lysine coated 24 well plates for 12–15 days. The cultures were treated with 10 μg/mL of the various RVG peptides and incubated for 1 h at 37°C. Subsequently, cells were fixated with 4% paraformaldehyde (PFA) in phosphate-buffered saline (PBS) for 10 min at room temperature, followed by three washes with PBS. Then, cultures were incubated with Streptavidin conjugated to Alexa Fluor 750 (for detection of the biotinylated peptides) and DRAQ5 (for detection of total cells in the culture; 1:1000, ThermoFisher, #62251) for 1 h. The cultures were then washed again with PBS and signals were measured by the ODYSSEY CLx scanner at 685nm and 785 nm (LI-COR Biosciences). Signals were quantified with the Image Studio software (LI-COR Biosciences) and normalized to DRAQ5 signals in the corresponding well.

### 2.6 Western blot analysis for testing peptide stability

For testing the resistance of the peptides to proteolytic degradation blood serum was prepared from blood collected from naïve mice decapitated under deep isoflurane anesthesia. The blood samples were allowed to clot at RT for 10–15 min, followed by centrifugation at 2000 *g* for 10 min and 4°C to separate the serum. The serum was stored at −80°C until used.

For testing, a peptide stock solution of 1 μg/μL was added directly to the serum in a ratio of 1:5 and incubated at 37°C for different times. After incubation, the mixture was diluted 1:4 with PBS and the samples were incubated with 2x Laemmli sample buffer (Bio-Rad) for 1 h at 37°C. The samples were subjected to tricine-sodium dodecyl sulfate-polyacrylamide gel electrophoresis (SDS-PAGE) using 1 mm thick 16% gels modified for the detection of small peptides as described by (Too et al., 1994).

Proteins were transferred onto 0.1 μm nitrocellulose membranes, previously coated with a solution of 0.5% gelatine (Too et al., 1994), in a semi-dry transfer cell (Trans-Blot SD; Bio-Rad) for 70 min at 10 V. After transfer, the blots were immediately incubated in 4% PFA for 5 min, thoroughly washed and blocked for 1 h in PBS containing 5% non-fat dry milk at room temperature. Thereafter, the blots were incubated with Alexa Fluor 750-streptavidin overnight at 4°C in PBS containing 5% non-fat dry milk. The blots were washed three times for 10 min with TBST and immunoreactivity was detected by the ODYSSEY CLx scanner (LI-COR Biosciences). For quantification of signal intensity, the images were analyzed with the Image Studio software (LI-COR Biosciences).

### 2.7 Immunofluorescence staining

The cell surface staining procedure for GABA_B_ receptors was carried out with living cultures to ensure intact plasma membranes and on ice with to prevent internalization of the receptors. For these experiments, antibodies directed against the extracellular located N-terminus of GABA_B2_ (GABA_B2N_) were used. Coverslips containing the cultured neuron/glia cells were washed 3 times for 5 min with cold buffer A (25 mM HEPES pH 7.4, 119 mM NaCl, 2.5 mM KCl, 2 mM CaCl_2_, 1 mM MgCl_2_ and 30 mM glucose). The primary antibody solution was prepared by diluting GABA_B2N_ antibodies 1:250 with a solution of 10% Normal Goat Serum (NGS) in buffer A. The coverslips were placed on parafilm on ice, and 60 µL of the antibody solution was applied onto each coverslip. The coverslips were incubated for 90 min on ice. After the incubation, the cover slips were washed three times with precooled buffer A for 5 min, followed by incubation with the secondary antibody diluted in 10% NGS in buffer A for 1 h on ice. After washing three times for 5 min with ice-cold buffer A, cells were fixated with 4% PFA in PBS for 30 min at room temperature. After fixation, the coverslips were rinsed once with PBS and then carefully dried and mounted upside-down onto glass slides using DAPI fluorescent mounting medium to visualize cell nuclei.

For standard immunofluorescence staining, the cells were briefly washed in PBS and then fixated with 4% PFA for 30 min at room temperature. After fixation, the cells were washed with PBS and permeabilized by incubation for 12 min in 0.2% Triton X-100/PBS. Then, the cells were incubated with primary antibody (diluted in PBS containing 10% NDS) overnight at 4°C. Next day, the coverslips were then washed 3 times for 5 min with PBS. Then, Alexa Fluor Plus secondary antibody (diluted in PBS/10% NDS) was added and incubated for 1 h at room temperature. The coverslips were then washed again 3 times for 5 min with PBS and mounted onto glass slides in DAPI fluorescent mounting medium to visualize cell nuclei.

To assess brain uptake of RVG-M1-Pep composed of D-amino acids, the biotinylated peptide (200 μg/mouse) was injected into the tail vein of mice. After 30 min, the brains were extracted, cut to separate the hemispheres, and immersed in 4% PFA for 1 h. The brains were then sectioned into 40 µm sagittal slices using a sliding microtome (HM400; Microm) and mounted onto SuperFrost Plus glass slides (Thermo Scientific). Following two 5-min washes with PBS, the sections were incubated for 1 h at room temperature with AlexaFluor 488-conjugated streptavidin (Jackson ImmunoResearch 016-540-084, 1:500) prepared in Tris-Triton solution (50 mM Tris, pH 7.4, 150 mM NaCl, 0.2% Triton X-100, and 5% NDS). After two additional 10-min washes with Tris-Triton, the sections were dried and cover-slipped using DAKO fluorescence mounting medium.

### 2.8 Glutamate-induced stress in neuron/glia cultures

To induce downregulation of GABA_B_ receptors and excitotoxic neuronal death in neuron/glia cultures, 1 mL of the culture medium from each coverslip-containing well was transferred to a fresh 12 well plate and stored in the incubator. The cultures in the original plate were then treated with 50 µM glutamate for 1 h. Subsequently the coverslips were transferred to the culture plate containing the saved original conditioned culture medium and incubated at 37°C and 5% CO_2_ for 16–24 h before being analyzed for cell surface expression of GABA_B_ receptors or neuronal death.

### 2.9 Quantification of neuronal death

Neuron/glia co-cultures were subjected to glutamate stress as described above and subsequently treated with 10 μg/mL of interfering peptides. After 24 h, the cultures were stained with an antibody directed against neuron-specific marker protein NeuN (1:400), followed by staining with Alexa Fluor Plus 488 labelled secondary antibodies (1:2000). The total number of cells were determined by staining the cell nuclei with DAPI included in the fluorescent mounting medium. After microscopy, the neuron and total cells were counted in the recorded images. The data are depicted as ratio of number of neurons over number of DAPI positive cells.

### 2.10 Confocal laser scanning microscopy and image analysis

**FIGURE 2:**
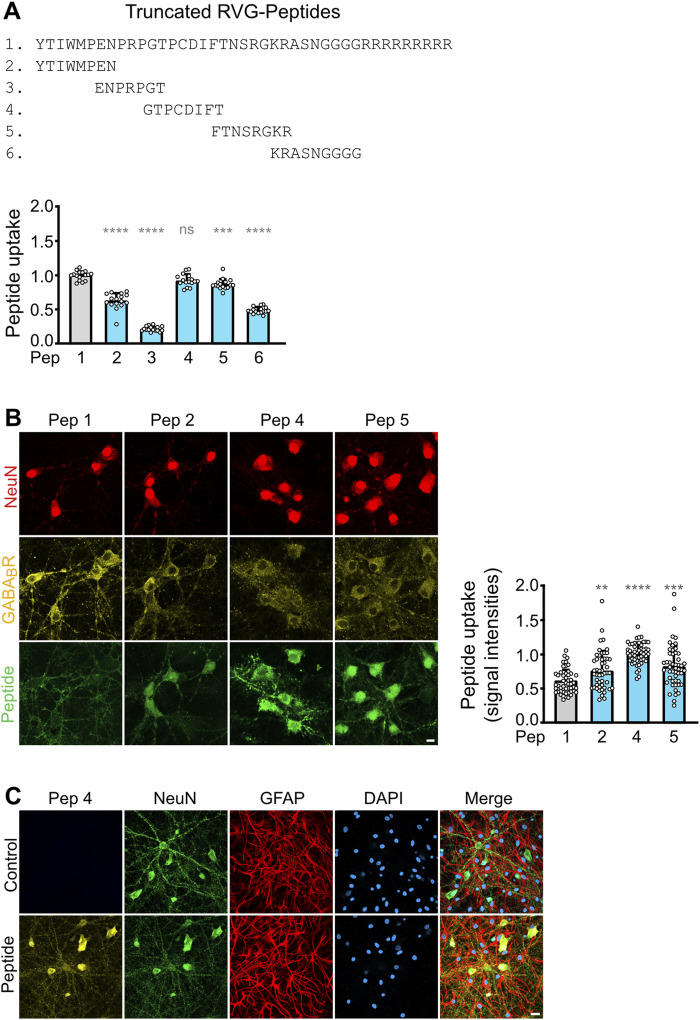
Optimization of the RVG peptide sequence composed of D-amino acids. **(A)** Screening for active sequences in the RVG peptide. The indicated truncated peptides (2–6) were tested for uptake into cultured neurons. The data was normalized to the signal of the original RVG sequence (peptide 1). The data represent the mean ± SD of the analysis of 16 neuronal cultures per sequence derived from two independent experiments. Brown-Forsythe and Welch’s one-way ANOVA followed by Games-Howell’s multiple comparisons test (ns, p > 0.05; ***, p < 0.001; ****, p < 0.0001). **(B)** Uptake of truncated, active RVG peptides by neurons. Neuron/glia cultures were incubated with the indicated peptides (green) for 1 h and then analyzed for colocalization with GABA_B_ receptors (yellow) and the neuron-specific marker protein NeuN (red). Left: representative images (scale bar: 10 μm). Right: Quantification of signal intensities. The number on the x-axis corresponds to the peptide number. For sequences see **(A)**. The original RVG sequence composed of D-amino acids (1) served as control. The data was normalized to the signal intensity of peptide 4 and represents the mean ± SD of the analysis of 50 neurons per sequence derived from two independent experiments. Brown-Forsythe and Welch’s one-way ANOVA followed by Games-Howell’s multiple comparisons test (**, p < 0.01; ***, p < 0.001; ****p < 0.0001). **(C)** Selective uptake of RVG peptide 4 into neurons. Neuron/glia cultures were incubated for 1 h with RVG peptide 4 (Peptide, yellow) or in the absence of peptide (Control) and then analyzed for colocalization with the neuron-specific marker protein NeuN (green) and the astrocytic marker protein GFAP (red). DAPI staining (blue) was used to detect all cells present in the culture (scale bar: 20 μm). The images shown are representative for 3 independent determinations performed in triplicates.

**FIGURE 3:**
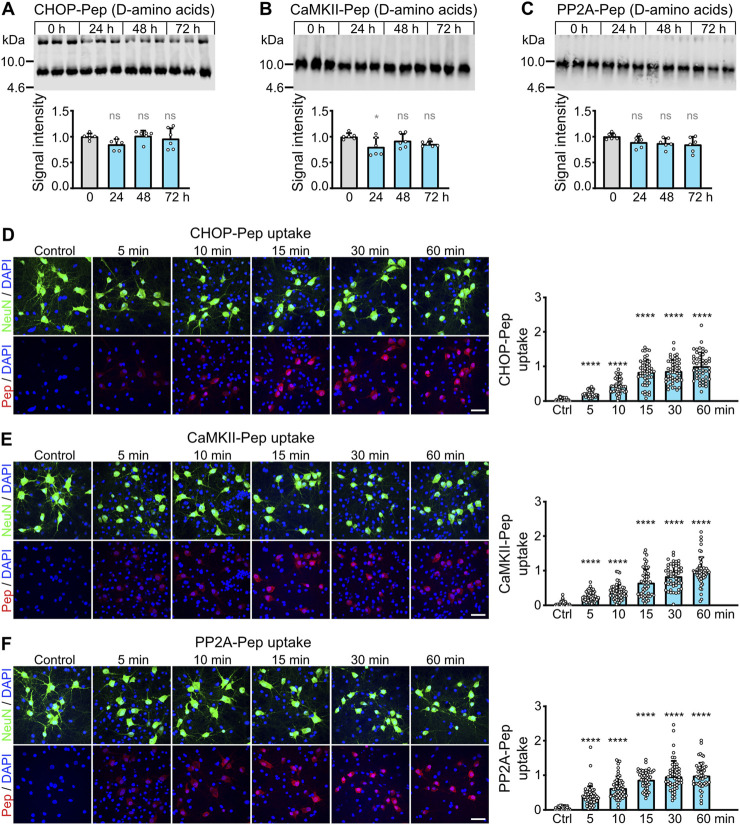
Optimization of CHOP-Pep, PP2A-Pep and CaMKII-Pep. **(A–C)** Proteolytic stability of the D-amino acid version of CHOP-Pep, PP2A-Pep and CaMKII-Pep containing the RVG peptide 4 sequence. Peptides were added to blood serum for the indicate times and analyzed thereafter by Western blotting. Top: representative Western blots. Bottom: Quantification of signal intensities. Time points 0 served as control. The data represent the mean ± SD of 3 independent experiments and one technical replicate. One-way ANOVA followed by Dunnett’s multiple comparisons test (ns, p > 0.05; *, p < 0.05). **(D–F)** Uptake of the D-amino acid version of CHOP-Pep **(D)**, CaMKII-Pep **(E)** and PP2A-Pep **(F)** containing the RVG peptide 4 sequence into cultured neurons. Neuron/glia cultures were incubated with the peptides for the indicated times and thereafter analyzed for the presence of the peptides (red), the neuron-specific marker protein NeuN (green) as well as total cells (DAPI, blue). Left: representative images (scale bar: 40 μm). Right: Quantification of signal intensities. The data was normalized to the 60 min time point and represents the mean ± SD of the analysis of 50 neurons per time point derived from two independent experiments. Brown-Forsythe and Welch’s one-way ANOVA followed by Games-Howell’s multiple comparisons test (****p < 0.0001).

**FIGURE 4:**
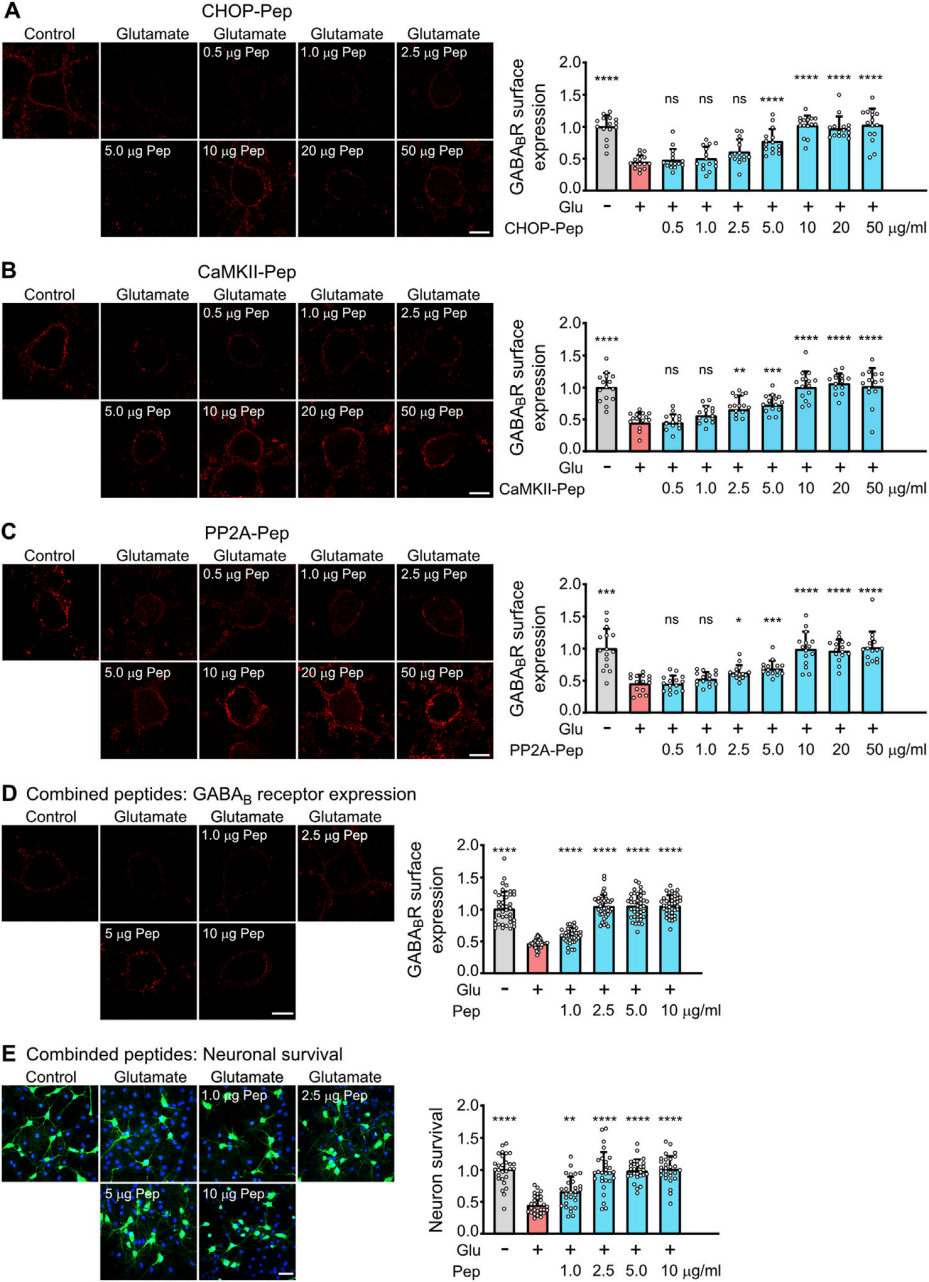
Evaluation of the dosage of the optimized interfering peptides to fully restore glutamate induced downregulation of GABA_B_ receptors **(A–D)** and to prevent excitotoxic neuronal death **(E)**. **(A–D)** Dosage of peptides. Neuron/glia cultures were subjected to glutamate stress (50 μM for 1 h) and subsequently incubated for 1 h with the indicated amounts (in μg/ml) of optimized CHOP-Pep **(A)**, CaMKII-Pep **(B)**, PP2A-Pep **(C)** or a combination of all three peptides **(D)**. The neurons were analyzed for cell surface expression of GABA_B_ receptors using antibodies directed against GABA_B2_ (red). Left: representative images (scale bar: 10 μm). Right: Quantification of signal intensities. The data was normalized to the untreated control and represents the mean ± SD of the analysis of 15 neurons per condition derived from two independent experiments. One-way ANOVA followed by Dunnett’s multiple comparisons test (ns, p > 0.05; *, p < 0.05; **, p < 0.01; ***, p < 0.001; ****p < 0.0001). **(E)** Neuroprotective activity of the combination of all three optimized interfering peptides. Neuron/glia cultures were subjected to glutamate stress (50 μM for 1 h) and subsequently incubated with the indicated amounts (in μg/ml) of a combination of all three peptides. After 24 h, cultures were analyzed for surviving neurons using staining for NeuN (green) and total cells (DAPI, blue). Left: representative images (scale bar: 40 μm). Right: Quantification of surviving neurons. The data was normalized to untreated control cultures and represents the mean ± SD of the analysis of 28 fields of view per condition derived from three independent experiments. One-way ANOVA followed by Dunnett’s multiple comparisons test (**, p < 0.01; ****p < 0.0001).

Images shown in Figures 1E, 2B,C, Figures 3D–F, 4A–E were taken with a Zeiss laser scanning confocal microscope (CLSM800 AiryScan) in sequential mode using the Zeiss 40x or 63x plan-apochromat oil immersion objectives (1.4 NA). Signal saturation was avoided by carefully adjusting the values of laser intensity and the detector gain. Images were recorded by sequential sampling across five z-planes, adhering to Nyquist sampling criteria. All images of one experiment were recorded in one continuous session with the same settings.

For quantification of signal intensities, the z-planes were merged into a single image for analysis using ImageJ. For determining cell surface staining, the outer and inner perimeter of the cell surface were exactly outlined. Then, the mean fluorescence intensity value obtained from the inner border was subtracted from the one of the outer borders so that only the fluorescence present at the cell surface was determined. For quantification of the total cell staining, only the outer border of the cell was marked, and the mean fluorescence intensity was measured.

### 2.11 Middle cerebral artery occlusion (MCAO)

Naïve C57Bl/6J mice were anesthetized with 5% isoflurane (Provet AG) and placed on a surgical setup, where anesthesia was maintained with 1.5%–2% isoflurane in a mixture of oxygen and air. Thirty minutes prior to surgery, mice received a subcutaneous injection of buprenorphine (0.1 mg/kg), and 5 min before starting a subcutaneous injection of lidocaine (5 mg/kg) and bupivacaine (1 mg/kg) at the site of incision.

Transient middle cerebral artery occlusion (MCAO) was performed as described previously (Hleihil et al., 2021). In brief, a midline neck incision was made to expose the left common carotid artery, and a 7–0 silicone-coated monofilament (Catalog No.: 701956PK5Re, Doccol Corp., Sharon, MA, United States) was inserted to induce unilateral MCAO. The artery was occluded for 60 min, after which the filament was removed to initiate reperfusion for 12 h. Peptides were injected intravenously (i.v.) immediately after starting reperfusion at a dose of 200 µg per mouse.

Following the procedure, mice were placed in a temperature-controlled recovery box at 37°C for 1–2 h before being returned to their home cages until further analysis. For sham-operated controls, the filament was briefly inserted to block the left middle cerebral artery and immediately withdrawn to allow instant reperfusion. The surgical steps for both groups were otherwise identical. To confirm successful MCAO surgery, the neurological deficits of mice were assessed using the Bederson score (Schaar et al., 2010) 1 h after reperfusion.

### 2.12 TTC (2,3,5-triphenyltetrazolium chloride) staining

About 12 h after MCAO surgery, mice were scarified by decapitation under deep isoflurane anesthesia. Brains were quickly removed, rinsed in cold phosphate-buffered saline (PBS) and sliced into 1 mm coronal sections using a mouse brain matrix. To visualize compromised tissue, the slices were immediately incubated in 1% TTC solution (prepared in PBS) at 37°C for 10 min. Following incubation, the sections were washed in PBS to remove excess TTC and photographed using a digital camera. Viable brain tissue was stained deep red, while infarcted areas remained unstained (pale white), indicating the lack of metabolic activity.

Two blinded experimenters quantified the infarct volume by measuring the pale, unstained areas in each brain slice using ImageJ (version 1.54). Sections of mice injected with the interfering peptides often displayed pinkish staining, which was classified as infarct tissue. The results of the two experimenters were averaged and displayed as a percentage of infarct volume of the ipsilateral hemisphere.

### 2.13 Statistics

The statistical evaluation of data was performed with GraphPad Prism (version 8.4.3). The data is given as mean value ±standard deviation (SD). One-way ANOVA was used to evaluate significant differences among conditions and was followed by appropriate *post hoc* tests as indicated in the figure legends. Data sets were tested for normal or lognormal distributions. In case of significant deviation from homoscedasticity, Welch and Brown Forsythe variations of ANOVA was used. A p-value of <0.05 was considered as statistically significant.

## 3 Results

### 3.1 Optimization of the neuron-specific RVG peptide for neuronal uptake and proteolytic stability

For rendering our interfering peptides cell permeable, we used the RVG peptide sequence, which specifically is taken up by neurons (Kumar et al., 2007; Balakrishnan et al., 2023; Hleihil and Benke, 2024). A simple way to protect peptides from proteolytic degradation is the substitution of natural L-amino acids with their unnatural D-enantiomers. As the inclusion of D-amino acids can compromise the activity of the peptide drug (Tugyi et al., 2005; Di, 2015), we systematically replaced L-amino acids in the RVG peptide sequence with the unnatural D-amino acids and assessed the impact on their uptake *in-vitro* into neuron/glia cultures (Figure 1A). Surprisingly, even completely changing the RVG peptide to D-amino acids (peptide 12) did not affect its uptake into the cultured cells.

We also tested whether the C-terminal added nine arginine (9R) to the RVG sequence are required for peptide uptake. Removal of the 9R sequence completely prevented the peptide uptake into cultured cells, demonstrating its necessity for cell penetration (Figure 1A, peptides 2–4).

Next, we tested the proteolytic stability of the RVG peptide completely composed of D-amino acids (peptide 12) versus the L-amino acid version (peptide 1). Western blot analysis demonstrated that about 60% of the L-amino acid version of the RVG peptide was degraded after 60 min, whereas the D-amino acid version remained stable (Figure 1B).

We then tested whether attaching a cargo peptide to the RVG peptide may alter the peptide stability. For this, we attached an interfering peptide (M1), which we recently developed to inhibit the interaction of GABA_B_ receptors with the E3 ubiquitin ligase MARCH1 (Bhat et al., 2025), to the RVG peptide. This longer peptide composed of L-amino acids was much more prone to proteolytic degradation than the RVG peptide without a cargo peptide. Already 5 min after incubation of the peptide in blood serum, most of the peptide was degraded (Figure 1C). However, the same peptide composed of D-amino acids remained stable over the time span tested (Figure 1C). The faster degradation of the RVG-M1 peptide versus the RVG peptide without cargo was not due to the specific M1 sequence, as the M1 peptide with a scrambled sequence showed the same time frame of proteolytic degradation (Figure 1D).

As expected, RVG-M1-Pep composed of D-amino acids injected into the tail vein of mice reached the brain as tested by immunohistochemistry (Figure 1E), verifying that RVG tagged peptides cross the blood brain barrier and are taken up by brain cells.

A major disadvantage of the RVG peptide as BBBpS is its length of 41 amino acids, resulting in very long and often difficult to synthetize peptides when cargo peptide sequences are attached. We therefore tested for sequences within the RVG peptide essential for its neuron-specific activity. Screening short overlapping peptide sequences derived from the RVG peptide identified three sequences that retained neuron-specific uptake when attached to the essential 9R sequence (Figure 2A, peptides 2, 4 and 5). These truncated peptides exhibited colocalization with GABA_B_ receptors and were selectively taken up by neurons, as evidenced by colocalization with the neuronal marker NeuN (Figure 2B). Quantification of peptide uptake into individual neurons by immunocytochemistry revealed that peptide 4 was considerably more efficiently taken up by neurons than the other peptides tested (Figure 2B). A further colocalization analysis confirmed the neuron-specific uptake of peptide 4 and lack of uptake by astrocytes, which is the predominant cell type in the neuron/glia cultures used (Figure 2C). We therefore used the optimized RVG peptide 4 composed of D-amino acids to render our interfering cell permeable in a neuron-specific manner.

### 3.2 Optimization of the stability of CHOP-Pep, PP2A-Pep, and CaMKII-Pep

Recently, we have developed three interfering peptides with neuroprotective activity *in-vitro* and *ex-vivo* after ischemic stress. These peptides restored downregulated cell surface expression of GABA_B_ receptor expression by preventing its interaction with CHOP (CHOP-Pep (Bhat et al., 2022)), PP2A (PP2A-Pep (Hleihil et al., 2022)) or CaMKII (CaMKII-Pep (Balakrishnan et al., 2023)). Here, we aimed to optimize the proteolytic stability of the interfering peptides for systemic *in-vivo* application. Based on our results on the RVG peptide (Figures 1, 2), we converted the interfering peptides into D-amino acids and tagged them with our newly identified RVG peptide 4 (also in D-amino acids) to render them cell permeable in a neuron specific manner. Incubation of these peptides for up to 72 h in mouse serum at 37°C did not result in an appreciable degradation of the peptides, highlighting their suitability for *in-vivo* applications with respect to their proteolytic stability (Figures 3A–C).

In the next series of experiments, we tested the time frame of peptide uptake into cultured neurons by colocalization immunocytochemistry. All three interfering peptides were rapidly and time-dependently taken up into cultured neurons. Already after 5 min, a considerable peptide uptake was detected, which reached the maximum after 15–30 min (Figures 3D–F). The peptides were specifically taken up by neurons as verified by colocalization with the neuron specific marker protein NeuN (Figures 3D–F).

We then analyzed the optimized peptides for their ability to restore cell surface expression of GABA_B_ receptors after excitotoxic stress and their neuroprotective activity. Exposure of neuron/glia cultures to excitotoxic stress for 1 h (50 μM glutamate) reduced cell surface expression of GABA_B_ receptors to about 50% (Figure 4). All three optimized interfering peptides dose-dependently restored the cell surface expression of GABA_B_ receptors in neurons after treatment with the different single interfering peptides or with a combination of all three peptides (Figures 4A–D). A peptide concentration of 10 μg/mL completely restored the cell surface expression of the receptors if cultures were treated with individual peptides (Figures 4A–C). However, a combination of all three interfering peptides was considerably more efficient than the single peptides. Already a concentration of 2.5 μg/mL of combined peptides completely restored cell surface expression of GABA_B_ receptor (Figure 4D). The higher efficiency of the peptide combination most likely results from an additive effect of targeting three distinct protein-protein interactions leading to the pathological downregulation of the receptors.

Finally, we analyzed the neuroprotective activity of the combination of all three peptides. Neuron/glia cultures were subjected to glutamate stress (50 µM) for 1 h and subsequently incubated with the increasing concentration of a combination of all three peptides. After 24 h, cultures were analyzed for surviving neurons by staining for neurons using the neuron-specific marker NeuN (Figure 4E). A peptide concentration of 2.5 μg/mL completely protected neurons from dying (Figure 4E). This peptide concentration matches nicely the concentration required to fully restore cell surface expression of GABA_B_ receptors (Figure 4D). Thus, the optimized interfering peptides fully retained their neuroprotective activity *in-vitro*.

### 3.3 *In vivo* neuroprotective activity of the optimized interfering peptides

**FIGURE 5:**
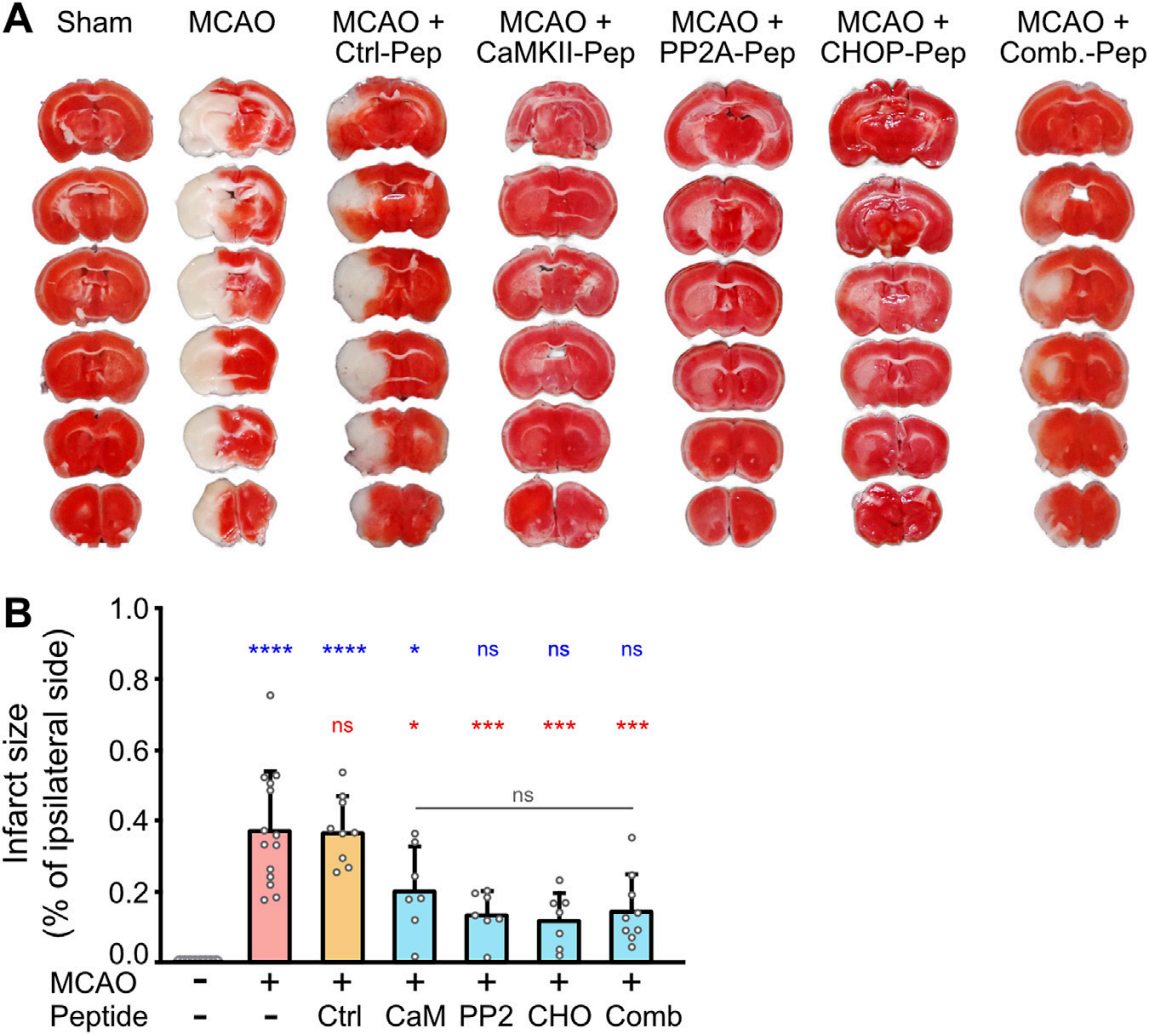
*In vivo* neuroprotective activity of optimized CaMKII-Pep, PP2A-Pep, CHOP-Pep and a combination of all three peptides (Comb.) in the MCAO mouse model of cerebral ischemia. Mice were subjected to MCAO for 1 h and then injected with the indicated peptides. After 12 h, brains were immediately sectioned into 1 mm slices and stained with TTC for living tissue (red). **(A)** Representative images. **(B)** Quantification of the infarct size of the ipsilateral side. Sham operated mice (--) served as a control for undamaged tissue after surgery without occlusion of the middle cerebral artery and injection of a control peptide (Ctrl-Pep) composed of random sequence served as a control for the peptide effect. The data represents the mean ± SD of 7–14 mice per condition. One-way ANOVA followed by Tukey’s multiple comparisons test (blue symbols: sham operated mice (--) vs. all other conditions; red symbols: MCAO vs. all other conditions; gray symbols: comparison within peptide conditions).

To evaluate the neuroprotective effects of our optimized interfering peptides *in-vivo*, we utilized middle cerebral artery occlusion (MCAO) as a mouse model of cerebral ischemia. MCAO was performed for 1 h, followed by intravenous injection of CHOP-Pep, PP2A-Pep, CaMKII-Pep, or the combination of all three peptides. Infarct sizes were assessed 12 h post-occlusion by TTC staining of 1 mm thick brain slices. Individually, CHOP-Pep, PP2A-Pep, and CaMKII-Pep reduced the infarct size by approximately 45%–70% compared to control mice injected with a random peptide sequence attached to the optimized RVG peptide (Ctrl-Pep) or untreated MCAO mice (Figures 5A,B). Although the combination of the three interfering peptides considerably reduced the infarct size by about 60%, it did not produce a stronger effect as compared to the injection of a single interfering peptide (Figures 5A,B). These results demonstrate the importance to protect peptide drugs from proteolytic degradation for their *in-vivo* activity after systemic application and verify *in-vivo* the neuroprotective potential of restoring downregulated GABA_B_ receptor expression after an ischemic insult.

## 4 Discussion

A major unresolved issue in the treatment of cerebral ischemia is the progressive death of neurons. Neuronal death spreading from the ischemic core to the surrounding brain tissue (ischemic penumbra) is mainly due to massive neurotransmitter release inducing sustained depolarizations, neuronal over-excitation and excitotoxicity. This longer lasting progressive neuronal death is considered amenable to neuroprotective treatments (Szydlowska and Tymianski, 2010; Bano and Ankarcrona, 2018; Choi, 2020).

GABA_B_ receptors are promising targets for the development of a neuroprotective intervention in cerebral ischemia as they are virtually expressed in all neurons in the brain to regulate their activity by conveying prolonged inhibition (Bettler et al., 2004; Chalifoux and Carter, 2011). One important physiological function of the receptors is to counteract over-excitation, which can otherwise lead to excitotoxic neuronal death. In addition, activation of GABA_B_ receptors triggers neuronal survival pathways and inhibits apoptotic pathways (Kuramoto et al., 2007; Cheng et al., 2010; Tu et al., 2010; Kim and Seo, 2013; Naseer et al., 2013; Fu et al., 2016; Li et al., 2017; Niu et al., 2017; Rai et al., 2019; Sun et al., 2020). However, under ischemic conditions, GABA_B_ receptors are downregulated by altered trafficking events, enhancing their lysosomal degradation (Guetg et al., 2010; Maier et al., 2010; Terunuma et al., 2010; Kantamneni et al., 2014; Maier et al., 2014; Zemoura et al., 2016; Zemoura et al., 2019). Because of their downregulation, the remaining GABA_B_ receptors do not have the capacity to counteract the massive neuronal overexcitation after an ischemic insult. Our recent research focused on the hypothesis that restoring GABA_B_ receptor expression and function by correcting the ischemia-induced dysregulated trafficking of the receptors will counteract overexcitation and protect neurons from dying. We concentrated on key GABA_B_ receptor interacting proteins mediating the downregulation of GABA_B_ receptors (CHOP, CaMKII and PP2A) and developed interfering peptides to inhibit their interaction with the receptors (for a review see (Bhat et al., 2023; Benke et al., 2024)). Treatment with the interfering peptides restored GABA_B_ receptor expression and function after an ischemic insult *in-vitro* and *ex-vivo* and stopped the progressive death of neurons (Bhat et al., 2022; Hleihil et al., 2022; Balakrishnan et al., 2023). However, they were ineffective after systemic treatment *in-vivo* most likely due to their fast proteolytic degradation in the circulation. The limited stability to proteolytic degradation is a major drawback of peptide drugs.

In this study, we optimized the three neuroprotective interfering peptides and the neuron-specific RVG peptide sequence for proteolytic stability to permit efficient systemic treatment *in-vivo*. A simple way to render peptides resistant to proteolytic degradation is the exchange of the natural L form of the amino acids with the unnatural D configuration (Tugyi et al., 2005; Di, 2015). However, this could alter the confirmation of the peptides and impact their biological activity. Surprisingly, we found that even after completely switching our peptides to D-amino acids, their activity remained unaffected. This suggests that the activity of the RVG peptide used for selective uptake into neurons as well as our three interfering peptides is solely sequence-dependent and does not rely on their conformation.

We found that the natural L-amino acid version of the RVG peptide was moderately stable in blood serum with about 60% of the peptide being degraded after 60 min. Unexpectedly, attaching a cargo peptide to the RVG sequence dramatically reduced the survival time to a few minutes. This was most likely due to the introduction of additional cleavage sites for proteases. In addition, the peptide length in general might also be a relevant factor as a scrambled version of the cargo peptide displayed the same behavior. However, the D-amino acid versions of our peptides were completely resistant to proteolytic degradation during the time span analyzed (up to 72 h).

We also optimized the RVG peptide sequence with respect to its length. The original RVG peptide is 41 amino acids long (Kumar et al., 2007), which gives rise to overly long and sometimes challenging to synthesize peptides when cargo sequences are added. We identified an eight amino acid long sequence within the RVG peptide that retains the information of neuron-specific targeting. In fact, this truncated version was considerably more efficiently taken up by neurons than the original RVG peptide. The combination of the optimized RVG peptide with our interfering peptides synthesized in the D-amino acid configuration were proteolytically stable, efficiently taken up specifically into neurons, restored GABA_B_ receptor expression in cultured neurons after excitotoxic stress and stopped progressive neuronal death. All these characteristics were required for *in-vivo* testing the optimized interfering peptides.

For analyzing the neuroprotective efficiency of the optimized interfering peptides *in-vivo* we used the MCAO mouse model. This model is based on the transient occlusion of the middle cerebral artery and closely mimics the pathological changes found in most human ischemic strokes (Sicard and Fisher, 2009). All three interfering peptides considerably reduced the infarct size to a similar extent confirming our previous *in-vitro* and *ex-vivo* data that restoring GABA_B_ receptor expression after an ischemic insult is neuroprotective. Although the combination of all three peptides showed a considerably higher potency in restoring GABA_B_ receptors and inhibiting neuronal death in cultured neurons, the peptide combination did not further reduce the infarct size in MCAO-treated mice beyond single peptide treatment. This indicates that the peptide concentrations used already exerted the maximum possible neuroprotective effect in the *in-vivo* experiments. It is very likely that considerably reducing the concentration of the combined interfering peptides will show an additive effect.

In contrast to our experiments on cultured neurons, we did not observe complete neuroprotection *in-vivo*. Complete neuroprotection in the MCAO model of cerebral ischemia is very unlikely even when the peptides are immediately injected after starting reperfusion because there will be immediate cell death in the ischemic core. In addition, the distribution and uptake of the peptides into the brain and neurons may take some time and delay the neuroprotective effect. We consistently observed areas with pink TTC staining in mice treated with the interfering peptides. These areas might indicate only slightly compromised but not fully damaged tissue. We included the areas with pink TTC staining in the infarct size determination, and therefore the infarct size in mice treated with the interfering peptides might be overestimated and the neuroprotective activity of the peptides underestimated.

There are several issues that need to be addressed in future experiments to fully explore the neuroprotective potential of our interfering peptides. First, the exact dosage of the interfering peptides to achieve their maximum neuroprotective effect needs to be determined. Second, the pharmacokinetics of the interfering peptides need to be analyzed. Are multiple injections required for maximum neuroprotection? Third, what are the long-term neurological benefits of peptide treatment in the recovery phase after the acute phase of cerebral ischemia? Fourth, what is the latest point for peptide treatment after the ischemic insult to achieve a beneficial neuroprotective effect?

In conclusion, we showed that optimizing interfering peptides for proteolytic stability is required for their effective *in-vivo* application. The presented data verified *in-vivo* that restoring the expression of GABA_B_ receptors after cerebral ischemia using interfering peptides to inhibit critical protein-protein interactions in the pathological pathways is a valid neuroprotective strategy.

## Data Availability

The datasets analyzed for this study will be deposited at ZENODO.org and are publicly available as of the date of publication (doi:10.5281/zenodo.15223089). Due to their large size raw images can be made available only upon reasonable request to the corresponding author.
